# Above and beyond C5a Receptor Targeting by Staphylococcal Leucotoxins: Retrograde Transport of Panton–Valentine Leucocidin and γ-Hemolysin

**DOI:** 10.3390/toxins9010041

**Published:** 2017-01-20

**Authors:** Gaëlle Zimmermann-Meisse, Gilles Prévost, Emmanuel Jover

**Affiliations:** Fédération de Médecine Translationnelle de Strasbourg (FMTS), VBP EA7290, Institut de Bactériologie, Université de Strasbourg, 3 rue Koeberlé, F-67000 Strasbourg, France; zimmermann.gaelle@gmail.com

**Keywords:** *Staphylococcus aureus*, C5aR binding leucotoxins, human neutrophils, confocal microscopy, retrograde transport, Fura-2 Calcium fluorimetry

## Abstract

Various membrane receptors associated with the innate immune response have recently been identified as mediators of the cellular action of *Staphylococcus aureus* leucotoxins. Two of these, the Panton–Valentine leucotoxin LukS-PV/LukF-PV and the γ-hemolysin HlgC/HlgB, bind the C5a complement-derived peptide receptor. These leucotoxins utilize the receptor to induce intracellular Ca^2+^ release from internal stores, other than those activated by C5a. The two leucotoxins are internalized with the phosphorylated receptor, but it is unknown whether they divert retrograde transport of the receptor or follow another pathway. Immunolabeling and confocal microscopic techniques were used to analyze the presence of leucotoxins in endosomes, lysosomes, endoplasmic reticulum, and Golgi. The two leucotoxins apparently followed retrograde transport similar to that of the C5a peptide-activated receptor. However, HlgC/HlgB reached the Golgi network very early, whereas LukS-PV/LukF-PV followed slower kinetics. The HlgC/HlgB leucotoxin remained in neutrophils 6 h after a 10-min incubation of the cells in the presence of the toxin with no signs of apoptosis, whereas apoptosis was observed 3 h after neutrophils were incubated with LukS-PV/LukF-PV. Such retrograde transport of leucotoxins provides a novel understanding of the cellular effects initiated by sublytic concentrations of these toxins.

## 1. Introduction

*Staphylococcus aureus* is a common constituent of the normal flora of the human body where it occurs in moist areas, such as nasal cavities, neck, or perineum, in roughly one-third of healthy adults. The prevalence of asymptomatic carriers overshadows the incidence of a broad variety of *S. aureus*-linked diseases [1], which range from minor infections of the skin to postoperative wound infections or highly threatening prosthetic resistant biofilms [2]. *S. aureus* strains are of uneven virulence and a higher pathogenic potential has long been associated with antibiotic resistance [3,4,5,6]. Nevertheless, the bacterial threat is also associated with the expression of particular virulence factors [7,8,9,10]. The genomes of antibiotic-sensitive and resistant *S. aureus* strains are highly variable, which increases the degree of bacterial hazard [11,12,13]. An examination of the relationship between virulence determinants in bacterial isolates and human disease suggests the necessity for matching factors between the two species to switch from asymptomatic carriage to disease; thus, expression of one particular virulence gene is not sufficient to predict virulence [14,15,16]. However, well-characterized staphylococcal secreted factors have been the object of particular attention as candidates for enhanced bacterial virulence [17,18,19,20,21]. Among them, leucotoxins form a family of secreted soluble beta-stranded proteins, which form pores in lipid membranes after seven identical monomers assemble into polymers [22] or by four dimers that organize into a complete octamer [23,24,25,26,27,28,29,30]. The two-component leucotoxins act through a synergistic association between a “slow-eluted” S compound (31–32 kDa), and a “fast-eluted” F compound (34–35 kDa) [31]. Five S and four F subunits affecting the human immune system have been described and they form the Panton-Valentine leucocidin LukS-PV/LukF-PV (PVL), the γ-hemolysins HlgA/HlgB and HlgC/HlgB, and the leucocidins LukA/LukB (or LukH/LukG) and LukE/LukD [32]. The S-subunit must bind to a membrane receptor to allow further association of the F-subunit and promote formation of the hetero-octameric complexes that subsequently form pores [33,34]. However, the functions ascribed to a particular secreted element according to in vitro assays may not accurately reflect behavior in vivo, as in infections produced from *S. aureus* PVL-producing strains, where the correlation with outcome severity remains controversial [35,36,37,38]. Although focusing on distinct toxins may be an oversimplification when considering *S. aureus* virulence, characterizing their cellular effects is of paramount importance due to the physiological impact of these pathogens on immune cells. Neutrophils are the main target of staphylococcal leucotoxins and a wide range of other bacterial factors. Neutrophils have been widely used in cellular studies, including modification of intracellular Ca^2+^ concentrations [39,40,41,42], oxidative burst [43,44], apoptosis [45,46], and neutrophil extracellular trap formation [47,48]. Various G-protein coupled receptors associated with innate immunity have been characterized as explicitly facilitating binding of the S-subunits. The LukS-PV and HlgC subunits bind to the C5a complement peptide receptor (C5aR), the HlgA subunit recognizes the chemokine receptors CXCR1/CXCR2 and CCR2, and the LukE subunit targets the CCR5 receptor [49,50,51,52,53]. However, the leucocidin LukA component (LukH) is an exception, as it targets human phagocytes by binding to CD11b, a component of Mac-1/CR3 integrin [25]. These results require closer scrutiny of leucotoxin-neutrophil interactions to consider an active role of the receptors in immune adaptation to *S. aureus* infection. Functional changes mediated through a receptor occupied by a leucotoxin may alter cell functions beyond the physicochemical multimeric subunit interactions thought to provoke cell lysis.

In a previous study, we characterized changes in free [Ca^2+^]_i_ induced by the PVL and γ-hemolysin HlgC/HlgB, which both act after binding of their respective S-subunit to the C5aR. Experimental evidence suggests that dissimilar internal stores act as sources of Ca^2+^ distinctly activated by HlgB (acidic stores) or LukF-PV (reticular stores) [39,41]. The cellular reaction to PVL or HlgC/HlgB binding to the C5aR differs from its response to the C5a peptide; therefore, we investigated whether the associations of leucotoxins with the receptor also differ from that of C5a in their intracellular pathway [41,54,55]. We found similar retrograde transport of the leucotoxins associated with the C5aR, but different kinetics were followed. The HlgC/HlgB-C5aR complex reached the Golgi network earlier than the PVL-C5aR complex. Moreover, neutrophils held the HlgC/HlgB intracellularly for up to 6 h without showing signs of cell death and the PVL for up to 3 h before the mitochondria depolarized and apoptosis was initiated.

## 2. Results

### 2.1. Leucotoxins Progress into the Cell in Association with the C5a Receptor Following Endocytosis

**Figure 1:**
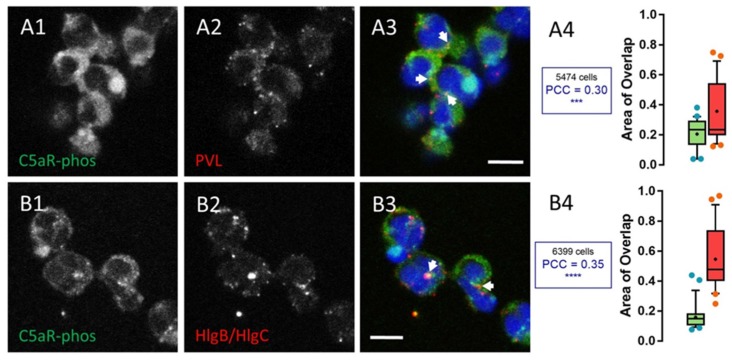
Both PVL and HlgC/HlgB are found with the phosphorylated C5a receptor in intracellular organelles. (**A1**–**A4**) Human neutrophils were incubated for 10 min with the PVL (0.25 nM), the toxin was removed, and the neutrophils were maintained at 37 °C for an additional 20 min. The cells were fixed and immunolabeled with: C5aR (**A1**); and LukS-subunit (**A2**) antibodies. (**A3**) A merged image of **A1** and **A2**. (**A4**) CellProfiler software was used to calculate Pearson’s correlation coefficient (PCC) between the two fluorescent markers. Values are compared with results of control cells, which were processed as experimental cells but in the absence of the leucotoxin. Box-and-Whisker’s plots show the relationship between the fluorescent labels by overlapping the labeled surfaces calculated with CellProfiler software. The green Box and Whiskers (median and percentiles) correspond to the percentage of total C5aR labeled area stained by the anti-leucotoxin antibody; the red Box is the percentage of the total surface labeled by the leucotoxin also stained with the anti-C5aR antibody. The number of cells considered is indicated above the PCC value. Arrows in the merged image indicate the points of most visible overlap between the two antibodies. (**B1**–**B4**) Human neutrophils incubated in the presence of 0.5 nM HlgC/HlgB. The results are presented as in (**A1**–**A4**) using CellProfiler software. Scale bars, 10 µm.

Binding of leucotoxin to human neutrophils increases free [Ca^2+^]_i_ initiated through their interaction with a membrane receptor. The PVL and HlgC/HlgB take advantage of the C5aR [52] and their [Ca^2+^]_i_ responses, which are distinguished by the time to peak of about 1 min for HlgC/HlgB and 5–6 min for the PVL, and by the identity of the internal compartments releasing Ca^2+^ [39,41]. In parallel, a complex comprised of a two-component leucotoxin associated with the receptor is removed from the membrane [41]. Deciphering the mechanism of this withdrawal is important to further understand the relationship between neutrophils and leucotoxins, particularly for the PVL, which does not modify resistance of the plasma membrane in the presence of physiological concentrations of Ca^2+^, as shown in our previous study [41]. The activated C5aR is known to be phosphorylated on its C-terminal region and internalized after binding the C5a complement-derived peptide [54,55]. We investigated the intracellular presence of leucotoxins presumably associated with the receptor using two monoclonal antibodies that recognize the same native or phosphorylated epitopes of the receptor (Figure S1). The cells were incubated for 10 min in the presence of the leucotoxin and further incubated for extended periods of time after removing the solution. Figure 1 shows an example of the uneven cellular distribution of the two leucotoxins 30 min after their initial binding.

The PVL-C5aR complex settled in a sub-plasmalemmal compartment (arrows, Figure 1A2,A3), whereas the HlgC/HlgB-C5aR complex transited to an area near the nuclei (arrows, Figure 1B2,B3). Pearson’s correlation coefficient (PCC) was determined for all acquired confocal optical slices [insets in Figure 1A4 (C5aR-PVL PCC = 0.30) and Figure 1B4 (C5aR-HlgC/HlgB PCC = 0.35)] to emphasize the presence of the leucotoxins and the phosphorylated C5aR in similar locations. Approximately 20% of the fluorescence associated with phosphorylated C5aR overlapped with the fluorescence associated with either leucotoxin at this point during the incubation. Similarly, more than 40% of the PVL-associated fluorescence and nearly 60% of the HlgC/HlgB-related fluorescence were found in an area also marked by C5aR-associated fluorescence. The fluorescence values associated with one channel overlapping the other from nearly 50 optical slices are presented as Box-and-Whiskers plots, indicating the median, quartiles, and interquartile range (Figure 1A4: PVL and 1B4: HlgC/HlgB). The differences in the size of the interquartile range indicate that the toxins accumulated in specific compartments also stained by the C5aR specific antibody. Moreover, the PCC was consistently positive and significant (*p* < 0.005) compared with the values calculated for control samples. Taken together, these results indicate that a significant proportion of the phosphorylated C5a receptor remains associated with structures also containing leucotoxins.

**Figure 2:**
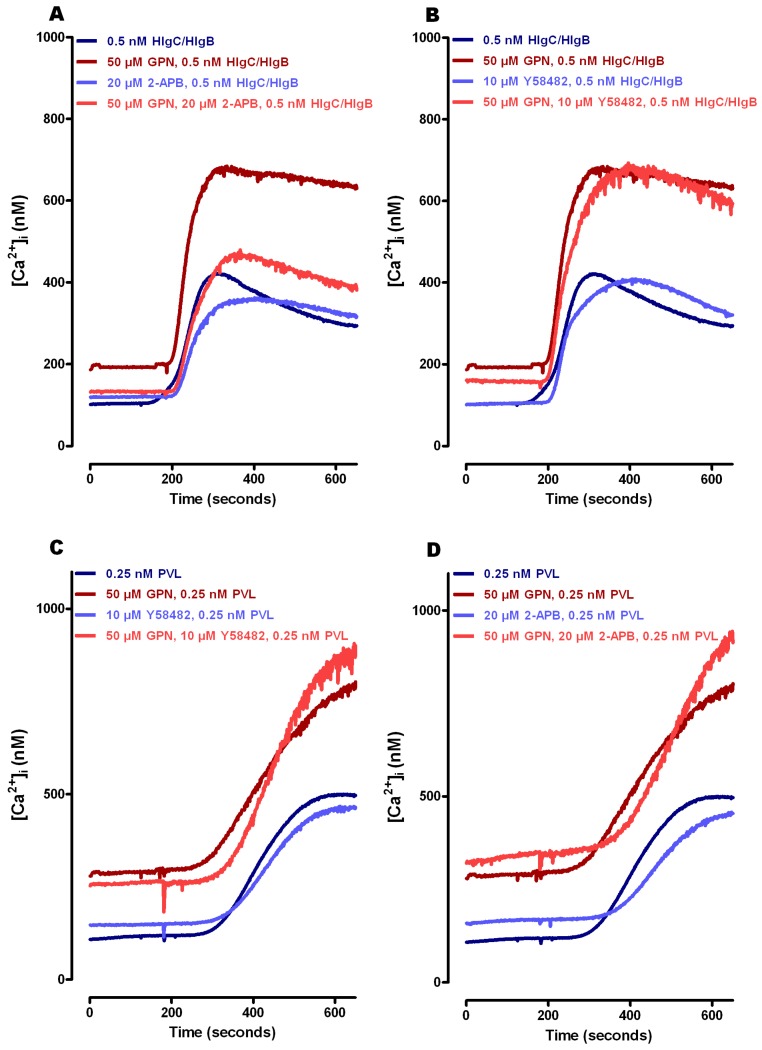
Leucotoxins require optimal buffer conditions for neutrophils to fully develop their activity. Human neutrophils recovered in a RPMI-10% FBS culture medium after purification were incubated for 1 h in 5 µM Fura-2 solution at 37 °C, washed by mild centrifugation and filtration, and maintained in the same buffer in the dark until use. (**A**) HlgC/HlgB-challenged neutrophils and the effect of blocking the store operated channels using 2-APB under control conditions and after disrupting the lysosomal compartment with GPN. (**B**) Effect of treating human neutrophils in the presence of YM 58483, which blocks the store operated channels, before challenge with 0.5 nM HlgC/HlgB as in (**A**). Human neutrophils from the same batches were used to analyze the effect of the PVL after incubation under identical conditions. Results are shown in (**C**,**D**). Traces represent the mean of a minimum of three independent experiments. The cells were incubated for 30 min in the presence of drugs (GPN, 26APB, and YM 58483), if needed, before the fluorescence recording. The toxins were added 180 s after starting to record.

The experiments were carried out using human neutrophils recovered in RPMI-10% fetal bovine serum (FBS) medium after purification under conditions aimed to preserve cell integrity and Ca^2+^ homeostasis. Then, we confirmed that the [Ca^2+^]_i_ responses and their pharmacology matched with published observations using cells temporally maintained in Ca^2+^ free-EGTA buffer [41]. We calculated the of Ca^2+^ concentrations from the Fura-2 fluorescence values using the Grynkiewicz equation [56]. Resting steady state free [Ca^2+^]_i_ was 108 ± 8 nM, which reached a maximum of 416 ± 39 nM within 90 s in the presence of 0.5 nM HlgC/HlgB, (Figure 2A,B).

Peak free [Ca^2+^]_i_ in the presence of 0.25 nM PVL was 475 ± 56 nM after >6 min (Figure 2C,D). Pre-treatment of the cells with the lysosomal disrupter glycyl-phenylalanine 2-naphthylamide (GPN) (50 μM) raised resting free [Ca^2+^]_i_ to 246 ± 29 nM. The higher free [Ca^2+^]_i_ contributed to a stronger increase of free [Ca^2+^]_i_ in cells challenged by HlgC/HlgB [∆ [Ca^2+^]_i_ + 37%] (Figure 2B) and in cells subjected to PVL [∆ [Ca^2+^]_i_ + 44%] (Figure 2D). Release of [Ca^2+^]_i_ from internal compartments of rat cerebellar neurons in response to the action of HlgC/HlgB activates store operated channels (SOCs) [39]. This effect can be pharmacologically blocked by YM 58483 (nicotinic acid adenine dinucleotide phosphate and SOC antagonist) or by the d-myo-inositol 1,4,5-trisphosphate receptor antagonist 2-APB, which also blocks particular transient receptor potential cation channels (TRP) [57,58]. The presence of 2-APB resulted in a 15% reduction in the free [Ca^2+^]_i_ peak due to HlgC/HlgB before GPN treatment and a 30% reduction after treatment (Figure 2A). The presence of YM 58483 also resulted in a 14% reduction in the HlgC/HlgB effect, but only in cells with preserved lysosomes (not exposed to GPN; Figure 2B). The increase in free [Ca^2+^]_i_ due to PVL was not associated with activation of a plasma membrane Ca^2+^ channel sensitive to 2-APB or to YM 58483 (Figure 2C,D), confirming previous observations [41]. Taken together, these results suggest the participation of an incoming Ca^2+^ pathway paired to SOCs, under the influence of HlgC/HlgB but not that of PVL.

### 2.2. HlgC/HlgB Quickly Reaches the Golgi Apparatus, While the PVL Transits through the Lysosomal System

The formation of pores in the lipid bilayer has been demonstrated for HlgC/HlgB but not for the PVL [59], and we previously reported that the PVL was unable to modify resistance of the plasma membranes of healthy cells under physiological conditions [41]. However, the particular intraluminal properties of cellular organelles may favor polymerization of leucotoxins and the formation of pores. Therefore, we identified cellular organelles where leucotoxins concentrated after internalization. We labeled early endosomes (anti-GTPase Rab5), recycling endosomes (anti-GTPase Rab11a), lysosomes (anti-lysosome-associated membrane glycoprotein 1, LAMP1), the endoplasmic reticulum (anti-protein disulfide isomerase, PDI), and the trans-Golgi network (TGN; anti-cation-independent mannose 6-phosphate receptor, CI-M6PR) using specific antibodies. Human neutrophils were incubated in the absence or presence of leucotoxins for different time periods and were labeled with both anti-toxin and anti-cellular organelle-specific antibodies. Samples of cells unchallenged by leucotoxins were processed as the experimental test cells and used as cell preservation controls to calculate PCC for randomly distributed fluorescence.

**Figure 3:**
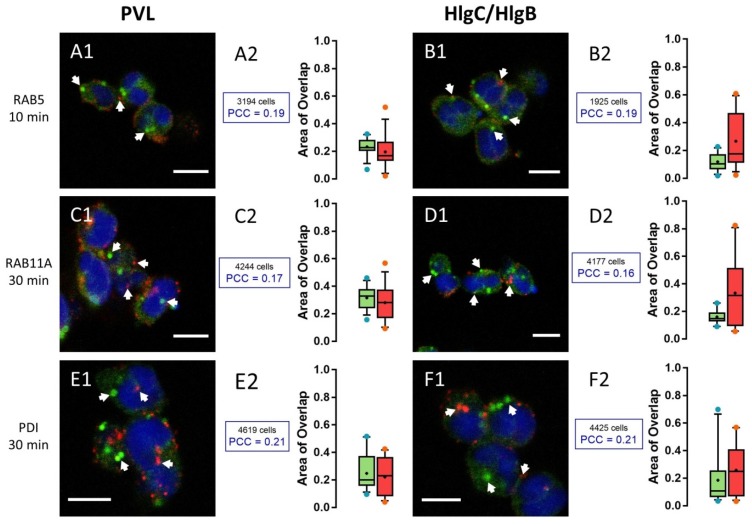
PVL and HlgC/HlgB leucotoxins do not remain in the early endosome (Rab5 labeling), the recycling endosome (Rab11b labeling), or the endoplasmic reticulum (PDI labeling). Examples of human neutrophils incubated with 0.25 nM PVL ((**A1,2**) 10 min; (**C1,2**) 30 min; and (**E1,2**) 30 min) or 0.5 nM HlgC/HlgB ((**B1,2**) 10 min; (**D1,2**) 30 min; and (**F1,2**) 30 min) and stained with antibodies against Rab5 (**A1**,**B1**), which concentrates in early endosomes. Labeling with anti-Rab11a antibody (**C1**,**D1**) highlights recycling endosomes, whereas the anti-PDI antibody (**E1**,**F1**) targets the endoplasmic reticulum. Arrows in each image indicate segregation between leucotoxin labeling and the three cell compartments. Overlap between the two markers can be observed in some cases, although the PCC values (**A2**–**F2**) for fluorescence co-distribution were low and not significantly different from control values, suggesting a random distribution. As in Figure 1, the Box-and-Whiskers plots (median and percentiles) are used to show the relationship between the fluorescent labels through overlap of the labeled surfaces. Green boxes indicate the values for the fraction of total surface labeled by: the anti-RAB5 antibody (**A2**,**B2**); the anti-RAB11A antibody (**C2**,**D2**); and the anti-PDI antibody (**E2**,**F2**) that was also labeled by the anti-leucotoxin antibody. Red boxes represent the percentage of total area labeled by the anti-leucotoxin antibody and stained by antibodies against the specific cellular compartments. The numbers of cells considered are indicated above the respective PCC values. In all cases, the percentage of surface labeled is compared with that of a control where the cells were processed with the same antibodies, but in the absence of leucotoxin. Scale bars, 10 µm.

Only 20% of the fluorescent signal due to the anti-Rab5a antibody overlapped with the fluorescence produced by anti-leucotoxin antibodies after a 10 min incubation of neutrophils with the PVL or HlgC/HlgB. The PCC values for the overlapping portion of the two labels (anti-Rab5 and a leucotoxin) revealed a random signal distribution (Figure 3A1,A2 for the PVL and Figure 3B1,B2 for HlgC/HlgB).

Very similar results were obtained for neutrophils stained with recycling endosomes (anti-Rab11a antibody) challenged with leucotoxins for various time periods. Cells incubated for 30 min are shown in Figure 3C1,C2 for the PVL and Figure 3D1,D2 for HlgC/HlgB. No specific co-distribution with the anti-leucotoxin antibodies is seen in the endoplasmic reticulum staining (anti-PDI antibody) after varying the incubation period. Figure 3E1,E2 shows neutrophils incubated for 30 min with the PVL prior to staining and Figure 3F1,F2 shows neutrophils after 30 min in the presence of HlgC/HlgB.

**Figure 4:**
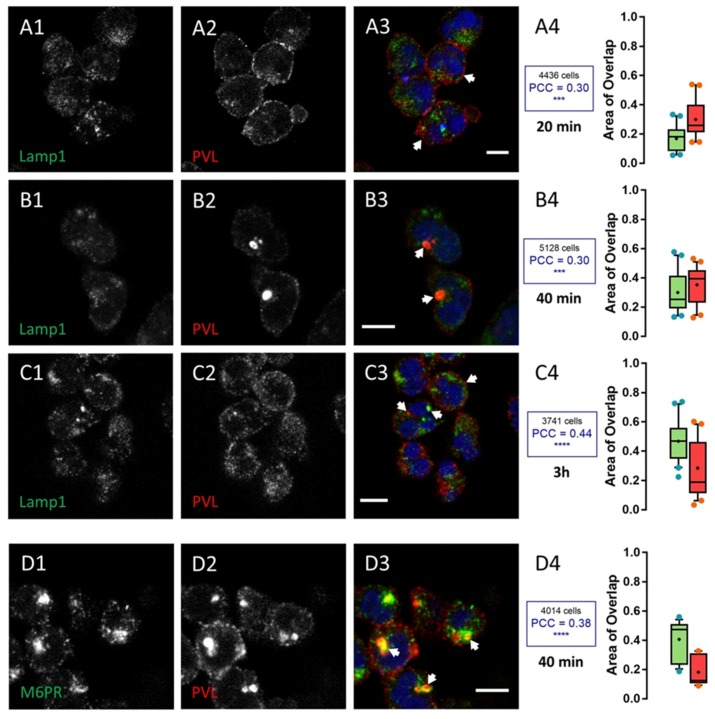
The PVL reaches the trans-Golgi network (TGN) 30 min after transiting through the lysosomal compartment. The human neutrophil lysosomal compartment was incubated with 0.25 nM PVL for: 20 (**A1**–**A4**); 40 (**B1**–**B4**); and 180 min (**C1**–**C4**) and immunostained with the anti-LAMP1 antibody. A significant proportion of the total surface labeled with the antibody is also associated with PVL-related fluorescence (arrows). Labeling was mainly concentrated in the area proximal to the nuclei. (**B1**–**B3**) The results after 40 min. (**D1**–**D3**) The TGN labeled with the anti-M6PR antibody after a 40 min incubation in the presence of the PVL. The Box-and-Whiskers plot shows the overlapping surfaces labeled by the two antibodies compared to the control. Red boxes show the percentage of total area labeled by the anti-leucotoxin antibody that is also stained by the other antibody. The number of cells considered in each case and the PCC for specific labeling are indicated in insets from (**A4**–**D4**). Scale bars, 10 µm.

**Figure 5:**
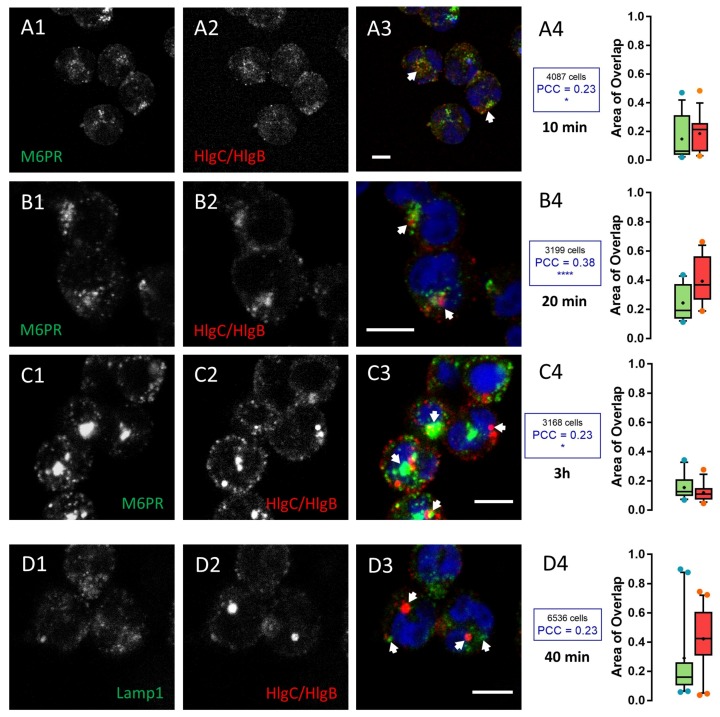
The HlgC/HlgB begins concentrating in the trans-Golgi network (TGN) 10 min after binding to the receptor. Human neutrophils incubated with 0.5 nM HlgC/HlgB for: 10 (**A1**–**A4**); 20 (**B1**–**B4**); and 180 min (**C1**–**C4**) were immunostained with the anti-M6PR antibody to highlight the TGN. Cells were processed as described in Figure 4. The results indicate that HlgC/HlgB began concentrating in the TGN after 10 min (**A1**–**A4**). A significant proportion of fluorescence emitted by the labels overlapped with the others, as shown in the Box-and-Whiskers plots. (**D1**–**D4**) An example of the segregation systematically observed after 40 min between the lysosomal compartment (stained by the anti-LAMP1 antibody) and intracellular localization of HlgC/HlgB. Scale bars, 10 µm.

Staining of lysosomes (anti-LAMP1 antibody) uncovered accumulation of the PVL (Figure 4), whereas staining of the TGN (anti-CI-M6PR) unmasked aggregation of HlgC/HlgB (Figure 5). Figure 4 shows human neutrophils incubated for 10 min with the PVL, then maintained for 20, 40, and 180 min before fixation and staining with the anti-LAMP1 antibody. About 30% of the surface labeled by the toxin was also labeled by the anti-LAMP1 antibody (red boxes in Figure 4A–C), whereas the proportion of area stained by the anti-LAMP1 antibody also labeled by the anti-leucotoxin antibody and increased slightly with time (green boxes in Figure 4A–C).

The PCC values revealed a significant non-random labeling distribution compared with control samples without toxin (*p* < 0.001 for 20 and 40 min and *p* < 0.0001 for the 3 h). The PVL began to concentrate in an area likely associated with the TGN (stained with anti-CI-M6PR) after a 40 min incubation with the toxin. About 40% of the anti-CI-M6PR staining significantly co-localized (*p* < 0.0001) with toxin-associated fluorescence (Figure 4D1–D4). However, HlgC/HlgB quickly transited to the TGN, as shown by the anti-CI-M6PR staining after a 10 min incubation with the toxin. Figure 5 shows HlgC/HlgB present in the TGN.

A 20 min incubation in the presence of leucotoxin resulted in 25% of the surface stained with the M6PR antibody, which was also stained by the anti-leucotoxin antibody (green box in Figure 5A4). In contrast, nearly 40% of HlgC/HlgB staining was detected over the surface corresponding to the TGN (red box in Figure 5A4, *p* < 0.0001). Values for the overlapping staining were similar after 40 min (Figure 5B1–B4, *p* < 0.001). However, co-distribution of anti-CI-M6PR and anti-HlgC/HlgB antibody staining decreased after 3 h in the presence of the leucotoxin (Figure 5C1–C4, *p* < 0.05), suggesting that the leucotoxin left the TGN. Staining for the HlgC/HlgB antibody never significantly overlapped with that of the anti-LAMP1 antibody. For example, neutrophils were stained after a 40 min incubation in the presence of HlgC/HlgB before fixation and labeling (Figure 5D1–D4). The concentrations of leucotoxins in the TGN have been confirmed by staining the Golgi apparatus with CTB-488 [60,61].

**Figure 6:**
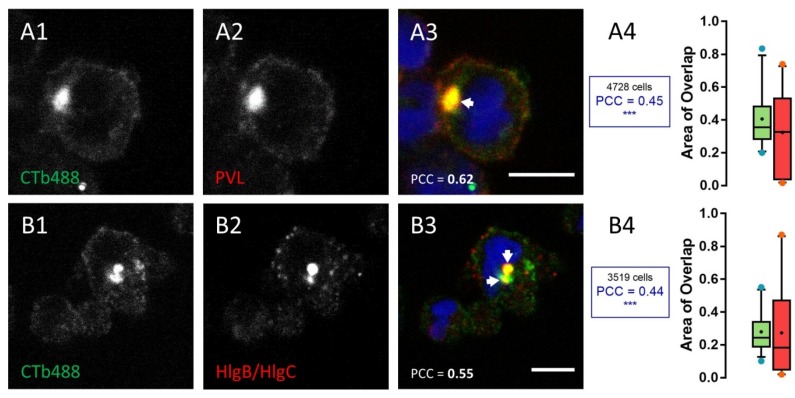
Co-localization of leucotoxins with the Cholera toxin b-subunit in the trans-Golgi network (TGN). Examples of human neutrophils incubated in the presence of: 0.25 nM PVL (**A1**–**A4**); or 0.5 nM HlgC/HlgB (**B1**–**B4**) for 40 min and then counterstained with the Alexa-488-derived b-subunit of the Cholera toxin, which binds GM1 gangliosides found in lipid rafts and subsequently concentrates in the TGN. (**A4**,**B4**) The overlapping percentage of surface labeled through Box-and-Whiskers plots. Scale bars, 10 µm.

Figure 6 shows examples of neutrophils incubated for 10 min at 37 °C in the presence of 0.25 nM PVL (Figure 6A1–A4) or 0.5 nM HlgC/HlgB (Figure 6B1–B4). After removing the leucotoxins, the cells were maintained for an additional 30 min at 37 °C in RPMI-FBS containing 2 µg/mL Alexa-488-CTB. Both leucotoxins were co-distributed with CTB, and a significant positive PCC was detected (*p* < 0.001) when compared to control neutrophils treated without leucotoxins. The HlgC/HlgB finding in the TGN early after internalization and in association with the C5a receptor (Figure 1) suggests that the latter drives leucotoxins [54]. On the other hand, the kinetics of PVL retrograde transport was slower than the kinetics of HlgC/HlgB transport.

Taken together, these observations suggest that leucotoxins do not modify permeability of the intracellular compartment membranes they pass through. The free Ca^2+^ in the cytosol after the PVL action is released from the endoplasmic reticulum [39,41], although the toxin is found early in the lysosomal compartment. The release of Ca^2+^ induced by HlgC/HlgB is from acidic stores [39,41], yet the toxin reaches the TGN during the early period of its activity.

### 2.3. Do Leucotoxins Modify the Life Span of Human Neutrophils by Remaining in Intracellular Compartments?

**Figure 7:**
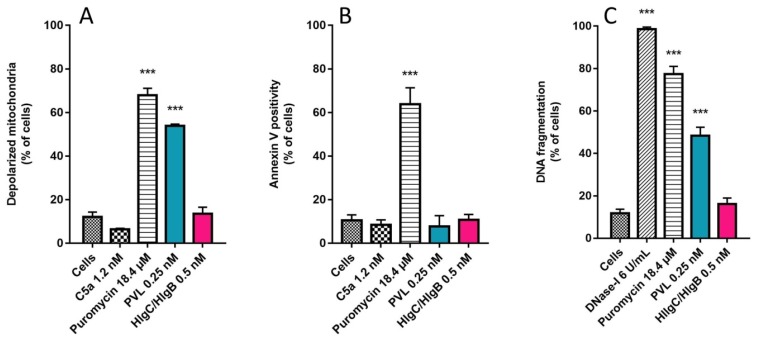
Human neutrophils overcame the intracellular presence of HlgC/HlgB for more than 6 h, whereas the PVL initiated apoptosis during this period. Three independent tests were used to estimate the initiation of apoptosis in human neutrophils incubated for 3 h in the presence of 0.25 nM PVL or for 6 h with 0.5 nM HlgC/HlgB by flow cytometry. (**A**) CCCP fluorescence associated with depolarized mitochondria showing 54% ± 1% of the PVL-treated cells compared to none of the HlgC/HlgB with labeling above background. (**B**) Annexin V labeling of externalized phosphatidylserine failed to highlight apoptotic human neutrophils treated with the PVL, whereas the TUNEL assay (**C**) confirmed that approximately 50% of PVL-treated cells were apoptotic. All three apoptosis detection protocols failed to reveal apoptotic activity in human neutrophils in the presence of HlgC/HlgB during the same time period.

The long-standing presence of the PVL and HlgC/HlgB in human neutrophils without causing any apparent damage is unexpected given the usual characteristic of staphylococcal leucotoxins as pore-forming molecules and the short half-life of neutrophils in the bloodstream [62]. Considering that these cells undergo apoptosis before being cleared by stromal macrophages [63], we used three approaches to assess likely early activation of apoptosis. The membrane-permeant 5,5,6,6-tetrachloro-1,1,3,3-tetraethylbenzimidazolylcarbocyanine iodide dye (JC-1) is widely used to monitor mitochondrial polarity. About 53% ± 5% of observed neutrophils incubated for 6 h in the presence of leucotoxins and 0.25 nM PVL had depolarized mitochondria, whereas none of the cells challenged for 6 h with 0.5 nM HlgC/HlgB had depolarized mitochondria (Figure 7A).

Untreated cells or cells incubated (6 h) in the presence of 1.2 nM C5a peptide were used as negative controls, whereas neutrophils challenged with 18.4 μM puromycin (68% ± 3% of cells analyzed) were the positive control [64]. Annexin V binding to externalized phosphatidylserine was also used to detect apoptosis. Neutrophils incubated in the presence of 18.4 μM puromycin were positive for Annexin V (64% ± 8%; Figure 7B), but cells incubated in the presence of PVL or HlgC/HlgB for 3 or 6 h were not. Fragmented apoptotic DNA was detected using a terminal deoxynucleotidyl transferase dUTP nick end labeling (TUNEL) assay after incubating neutrophils for 6 h with puromycin (77% ± 4%) or 0.25 nM PVL (48% ± 4%). No fragmented DNA was detected in the neutrophils incubated with 0.5 nM HlgC/HlgB (Figure 7C).

## 3. Discussion

A primary aim of this study was to suggest the fate of staphylococcal leucotoxins after binding to the C5aR on human neutrophils and not altering the resistance of the plasma membrane of healthy cells while eliciting an increase in free [Ca^2+^]_i_ [41]. We and others previously supported the notion that neutrophils in vitro can overcome the presence of low staphylococcal leucotoxin concentrations, provided that the experiments are carried out using physiological concentrations of extracellular Ca^2+^ [39,41,44]. Although the test proposed by our colleagues Fink-Barbançon and Gauduchon et al. [65,66,67] to determine pore formation, which only works in the absence of extracellular Ca^2+^, is widely accepted, neither we nor others have demonstrated that ethidium bromide or propidium iodide cross the plasma membrane through a pore formed by the PVL. This observation indicates that in vitro data do not exactly match the consequences of infection, as suggested by the inverse association between cytotoxicity and mortality [10]. Indeed, use of particular physicochemical conditions, including high concentrations of leucotoxins, has generated valuable results regarding the interaction between subunits in lipid bilayers and their crystalline structure [68,69,70,71]. However, a holistic explanation about the cellular activity of leucotoxins must consider the functional consequences of identifying specific cellular receptors for leucotoxins [25,49,50,51,52,53]. Moreover, previous observations from our laboratory and others [39,40,41,42,43,44,45,46,47,48] are better understood by considering active participation of these receptors in cellular leucotoxin activities. In the present study, maintaining human neutrophils in RPMI-10% FBS medium improved cell survival and supported vigorous intracellular Ca^2+^ responses. Accordingly, estimates of [Ca^2+^]_i_ were obtained in resting and activated neutrophils based on the Fura-2 fluorescent signal (Figure 2) [56,72]. Additionally, the neutrophil responses were better estimated in cells challenged by leucotoxins under various pharmacological conditions (Figure 2). Our present HlgC/HlgB activity data agree with previous observations on neurons [39] and suggest activation of a plasma membrane Ca^2+^ channel in neutrophils that was not expressed by neurons. A likely candidate is TRPM2, which is also sensitive to 2-APB [57,58]. Notably, PVL action in neutrophils in the presence of GPN revealed a source for release of [Ca^2+^]_i_ other than the lysosomes, as neutrophils responded similarly to PVL stimulation (amplitude and time to maximum) of control cells and cells with a disrupted lysosomal system (Figure 2).

We have previously suggested that internalization of leucotoxin components (S- and F-subunits) associated with C5aR may precede mobilization of [Ca^2+^]_i_ [41]. After partially challenging this hypothesis, we confirmed internalization of the two leucotoxins and the receptor but failed to demonstrate endocytosis as the primary mechanism for mobilizing free [Ca^2+^]_i_. Comparing the kinetics of Ca^2+^ mobilization with those of retrograde transport will not help uncover the initial step in the process. Moreover, the intracellular locations of the leucotoxins do not suggest release of Ca^2+^ through a pore formed in the compartment. The PVL accumulated in the lysosomal compartment, but induced Ca^2+^ release from the ER, and HlgC/HlgB reached the TGN early but provoked Ca^2+^ release from acidic compartments. Previous studies suggested that the PVL may control oxidative burst [43] and alter gene expression [44] in human neutrophils. The agonist-mediated internalization of the C5aR is modulated by phosphorylation of the C-terminal domain [54]. Here, we found that the two leucotoxins internalized with the phosphorylated form of the receptor (Figure 1). Furthermore, binding of LukS-PV was regulated by protein kinase C [66]. Intriguingly, LukS-PV and HlgC do not bind the C5aR through the same interactions [41,52]. Such a disparity may be part of the gap between the actions of the two leucotoxins. Formation of the dimer requires binding of the S-subunit to the receptor, which may justify the preferred associations between HlgC with HlgB and that of LukS-PV with LukF-PV. Moreover, the high affinity binding site for LukF-PV described previously [73] and the increase in intracellular Ca^2+^ generated by the associations between Luk-S-PV with HlgB or HlgC with Luk-F-PV [41] suggest other elaborate interactions with unknown cell components. Retrograde transport of the leucotoxins associated with the C5aR is roughly the same as transport initiated by C5aR after binding C5a [54,55]. These similarities suggest a route that facilitates the intracellular activities of the leucotoxins and provides cues to uncover other partners for their actions. The prolonged survival of cells loaded with leucotoxins (Figure 7) before initiation of the apoptotic pathway was verified with three tests of increasing sensitivity, and the results were consistent with the half-life of circulating neutrophils and their degradation [63]. Such a long survival duration fits with the global changes in gene expression after the 3 h treatment with a low PVL concentration [44] and is consistent with other cellular effects [47,48].

Is cytotoxicity induced in vitro in the absence of Ca^2+^ more threatening than any effect observed at a low concentration in vivo? Significant levels of circulating antibodies are detected in infected individuals [74,75] but no available data suggest the leucotoxin concentrations in blood, even though extremely high quantities of leucotoxins are detectable in skin, respiratory tract, and joint fluid from infected individuals [76]. The initial rise of free [Ca^2+^]_i_ evoked by low concentrations of leucotoxins bound to the C5aR is sufficient to activate neutrophils. Ca^2+^-induced mobilization and internalization of the receptor-toxin complex could be more of a diversion of the initial function of neutrophils rather than a true immune response. Then, the absence of this neutrophil function and their priming for other cellular functions could facilitate bacterial replication. In addition, cells from tissues that also express the C5aR would be targeted by leucotoxins, which increases the *S. aureus* threat. The notion that a host factor predisposes severe disease related to *S. aureus* is emerging [10,14,15,77]. This is a compelling argument for a careful consideration of the molecular interactions between bacterial factors and the first host cells exposed. Finally, we must consider that the affinity of leucotoxins for their receptors is not equal in all species and that differences in the responses of the receptors cannot be excluded depending on the laboratory animal model.

## 4. Materials and Methods

### 4.1. Ethics Statement

Buffy coats, purchased from the “Établissement Français du Sang (Strasbourg, France),” were from adult volunteers who provided informed consent. Written consent was collected by the Établissement Français du Sang, which maintained confidentiality of the donor information.

### 4.2. Drugs, Chemicals, and Antibodies

Glycyl-1-phenylalanine 2-naphthylamide (GPN), puromycin, and Triton X-100 were purchased from Sigma-Aldrich (Saint-Quentin Fallavier, France). Blockers of store-operated Ca^2+^ entry, such as *N*-[4-[3,5-*Bis*(trifluoromethyl)-1*H*-pyrazol-1-yl]phenyl]-4-methyl-1,2,3-thiadiazole-5-carboxamide (YM 58483), and glycyl-phenylalanine 2-naphthylamide (GPN) and the uncoupler of oxidative phosphorylation carbonyl cyanide m-chlorophenyl hydrazine (CCCP) were obtained from Tocris Bioscience (Bristol, United Kingdom). Fura-2 acetoxymethyl ester (Fura-2/AM) was purchased from Molecular Probes/Life Technologies (Fisher Scientific, Illkirch, France). Primary mouse monoclonal antibodies against human C5aR-C-terminal phosphorylated (p-CD88 32-G1; sc-53793), LAMP1 (H4A3; sc-20011), Rab11a (D-3; sc-166523), Rab5 (D-11; sc-46692), and protein disulfide-isomerase (PDI, C-2; sc-74551) were obtained from Santa-Cruz Biotechnology (Heidelberg, Germany). The anti-M6PR (cation independent) antibody (2G11; ab2733) was purchased from Abcam (Cambridge, UK) and JC-1 [5,5,6,6-tetrachloro-1,1,3,3-tetraethylbenzimidazolylcarbocyanine iodide] fluorescent dye, an indicator of mitochondrial membrane potential, was obtained from ATT Bioquest (Souffelweyersheim, France).

### 4.3. Preparation of Human Polymorphonuclear (hPMN) Cells

Human PMNs were prepared from buffy coats. The cells were prepared within 24 h after blood donation, according to a procedure described previously [65,73]. hPMNs were resuspended in RPMI media supplemented with 10% FBS after purification.

### 4.4. Leucotoxin Purification

The *S. aureus* HlgC/HlgB and the PVL LukS-PV/LukF-PV were purified as described previously [66,78] by affinity chromatography on glutathione-Sepharose 4B followed by cation-exchange fast-performance liquid chromatography after removing the GST tag with Precision Protease (GE Healthcare, Villacoublay, France). Preparation homogeneity was assessed by radial gel immunoprecipitation and SDS-polyacrylamide gel electrophoresis before storage at −80 °C [66].

### 4.5. Spectrofluorimetry

Variations in intracellular free Ca^2+^ levels were determined by recording the Fura-2 fluorescence contained in hPMNs, as described previously [65]. Briefly, neutrophils were loaded with 4 µM Fura-2 AM in EGTA buffer for 45 min in the dark at room temperature. The hPMNs were washed twice and then suspended (3.5 × 10^6^ cells/mL) in EGTA buffer. Two milliliters of hPMNs were incubated for 5 min with 1.1 mM CaCl_2_ in a 4-mL polystyrene cuvette (1-cm light path). Changes in fluorescence intensity were recorded at 37 °C with a dual-excitation spectrofluorometer (Deltascan; PTI, Houston, TX, USA) operated in Fura-2 ratio mode at excitation wavelengths of 340 and 380 nm (slit width, 4 nm) and an emission wavelength of 510 nm (slit width, 4 nm). The fluorescence intensity ratio (R = Ex340 nm/Ex380 nm) was calculated as arbitrary fluorescence units for each pair of wavelengths determined. Calcium concentration was estimated by the following formula [56]:

$${\left[{\mathrm{Ca}}^{2+}\right]}_{i}=K_{d}\times \beta \times (R-R_{min})/\left(R_{\max}-R\right)$$

where *K*_d_ is the dissociation constant of Fura-2 for Ca^2+^ (340 nM, [56]), and *β* = (*I_380 max_*)/(*I_380 min_*) (3.57 ± 1.34). The *R_min_* and *R_max_* values were determined for the regular experimental series.

Rmin and Rmax were determined after permeabilizing the cells in buffer containing 10 µM A23187 complemented with 0.1/1000 (*v/v*) Triton X-100. The Fura2 fluorescence was recorded for 30 minutes, or until the calcium concentrations of the two compartments were equilibrated. The values recorded in EGTA-Ca^2+^ free buffer were used to determine the Rmin and values recorded in the usual HBSS buffer to determine Rmax.

### 4.6. Mitochondrial Membrane Potential (Δψm) Estimates

To assess Δψm of neutrophils, 10^6^ cells/mL in RPMI-10% FBS were incubated with either toxin. The cells were stained with JC-1 fluorescent dye (5 µM) for 10 min at room temperature after 3 and 6 h incubations at 37 °C. Positive controls for mitochondrial membrane depolarization were obtained by incubating the cells in a 10 µM CCCP solution for 15 min. Fluorescence intensities were estimated on a FACSort cytometer (Becton Dickinson, Le Pont de Claix, France) equipped with a 15-mW argon laser tuned to 488 nm by recording through the FL1 channel (emission wavelength, 530 nm) and the FL2 channel (emission wavelength, 585 nm). Cell Quest software was used to approximate the percentage of cells analyzed in both populations: the polarized mitochondrial membrane was observed as orange fluorescence and the depolarized membrane as green fluorescence.

### 4.7. Assessment of Neutrophil Apoptosis by Annexin-V Binding and TUNEL Assays 

The percentage of apoptotic neutrophils was determined according to the manufacturer’s specifications Santa-Cruz Biotechnology (Heidelberg, Germany) using Annexin V-FITC, which binds phosphatidylserine. Cells (10^6^ cells/mL in RPMI-10% FBS) were incubated with either of the two toxins. After 3, 6, or 20 h incubation at 37 °C, the cells were centrifuged, and the pellet was resuspended and stained by adding 0.1 µg Annexin V and 1.3 µg/mL propidium iodide for 10 min at room temperature. The analysis was performed using flow cytometry. The TUNEL assay was used to observe DNA fragmentation in accordance with the manufacturer’s specifications (Roche, Bâle, Switerzland). DNase I (Sigma-Aldrich (Saint-Quentin Fallavier, France) was used as a positive control at 6 U/mL. The cells were incubated in the presence of leucotoxins for 6 h at 37 °C (2 × 10^6^ cells/mL, RPMI-10% FBS) and then fixed for 10 min at room temperature in 4% (*v/v*) paraformaldehyde Hank's Balanced Salt Solution in 20 mM HEPES buffer (HBSS-HEPES). After washing with HBSS-HEPES, the neutrophils were permeabilized in the same buffer containing 0.05% Triton X-100, washed, and incubated in the TUNEL mixture for 1 h at 37 °C. The cells were centrifuged, the pellet was resuspended in HBSS-HEPES, and the number of labeled cells was determined by flow cytometry.

### 4.8. Immunocytochemistry

Rabbit polyclonal antibodies independently raised against the HlgC, HlgB, LukS-PV f(ab’)^2^, and LukF-PV subunits [73,79] were used to detect the cellular location of leucotoxins in human hPMNs. Diverse intracellular compartments were labeled using monoclonal antibodies raised against Rab5 (early endosome, sc-46692, Santa Cruz Biotechnology), Rab11a (recycling endosome, sc-166523, Santa Cruz Biotechnology, Heidelberg, Germany), LAMP1 (lysosome), PDI (endoplasmic reticulum, sc-74551, Santa Cruz Biotechnology, Heidelberg, Germany), M6PR (TGN/lysosome, ab2733, Abcam, Cambridge, UK), and Alexa-488-labeled Cholera toxin B-subunit (Golgi apparatus, Life Technologies, Carlsbad, CA, USA). Cells, (8 × 10^6^ cells/mL in RPMI-10% FBS) were maintained for 10 min at 37 °C, in a 5% CO_2_ incubator with 0.25 nM of the PVL or 0.5 nM of HlgC/HlgB. The toxins were removed by centrifugation (2100× *g*, 1 min), resuspended in fresh medium, and deprived of toxins for further incubation (10, 20, 30 min, and 3 h). After each incubation period, the cells were fixed for 10 min in 4% (*v/v*) paraformaldehyde- HBSS-HEPES and maintained for 30 min in blocking buffer (10% FBS, 5 mg/mL bovine serum albumin [BSA] in PBS) at room temperature after washing. The cells were permeabilized by a 5 min incubation in 0.05% Triton X-100 containing HBSS-HEPES, and then washed before the adding the antibodies. Cells and primary antibodies were diluted 1 μg/mL in HBSS-HEPES containing 5% FBS and 1 mg/mL BSA and were maintained overnight at 4 °C. After two washes, the cells were incubated with DyLight labeled secondary antibodies (Bethyl, Souffelweyersheim, France) for 60 min, washed, and incubated for 15 min in the presence of 10 μg/mL Hoechst 32258 (Sigma-Aldrich) for labeling of cell nuclei. The pellets were resuspended in Mowiol coverslip mounting solution (Mowiol 4.88; cat# 475904; Calbiochem, La Jolla, CA, USA), mounted on slides, and stored at 4 °C until observation on a Leica SP5-II inverted confocal microscope (63× objective). Fields of 1024 × 1024 pixels were acquired using a 305 nm diode (ultraviolet), a 488 nm argon laser, and a 561 nm diode-pumped solid-state laser.

### 4.9. Image Analysis

Confocal microscope-acquired images were analyzed with CellProfiler software ver. 2.2 for Windows (Brod Institute, Harvard, Cambridge, MA, USA) [80] which allows the determination of PCC and the overlap ratio with D488 (DyLight 488) and D594 (DyLight 594) images. Results are obtained by a sequence of modules forming a pipeline, both of which are described and can be downloaded at: http://cellprofiler.org/published_pipelines.html. PCCs were determined on full images without cell segmentation after removing cellular debris images, in which a threshold was used to determine a background on cells with a PCC = 0.1. Percentage overlap was determined using segmentation to establish the D488 and D594 spots in each channel; the process does not include cellular debris-associated light, and the two ratios were calculated based on the area measurement.

### 4.10. Statistical Analysis

Results are expressed as mean ± standard error of the mean of at least three independent experiments. GraphPad Prism ver. 5 for Windows software (GraphPad Software, La Jolla, CA, USA) was used to calculate the means for each experimental condition, prepare the graphs, and perform the statistical analysis. A two-way analysis of variance followed by a Bonferroni post-test was used to detect differences between experimentally treated and control cells. A *p*-value < 0.05 was considered significant.

## Supplementary Materials

The following are available online at www.mdpi.com/2072-6651/9/1/41/s1, Figure S1: Binding of the PVL or HlgB/HlgC induces phosphorylation of C5aR.
