# Supplementary material for: Above and beyond C5a Receptor Targeting by Staphylococcal Leucotoxins: Retrograde Transport of Panton–Valentine Leucocidin and γ-Hemolysin
Source: Toxins (Basel). 2017 Jan 20;9(1):41. doi: 10.3390/toxins9010041 (PMC5308273; doi:10.3390/toxins9010041)
Supplement: Supplementary file 1 [file toxins-09-00041-s001.pdf]

Supplementary Materials: Above and beyond C5a Receptor Targeting by Staphylococcal Leucotoxins: Retrograde Transport of Panton–Valentine Leucocidin and γ-Hemolysin

Gaëlle Zimmermann-Meisse, Gilles Prévost and Emmanuel Jover

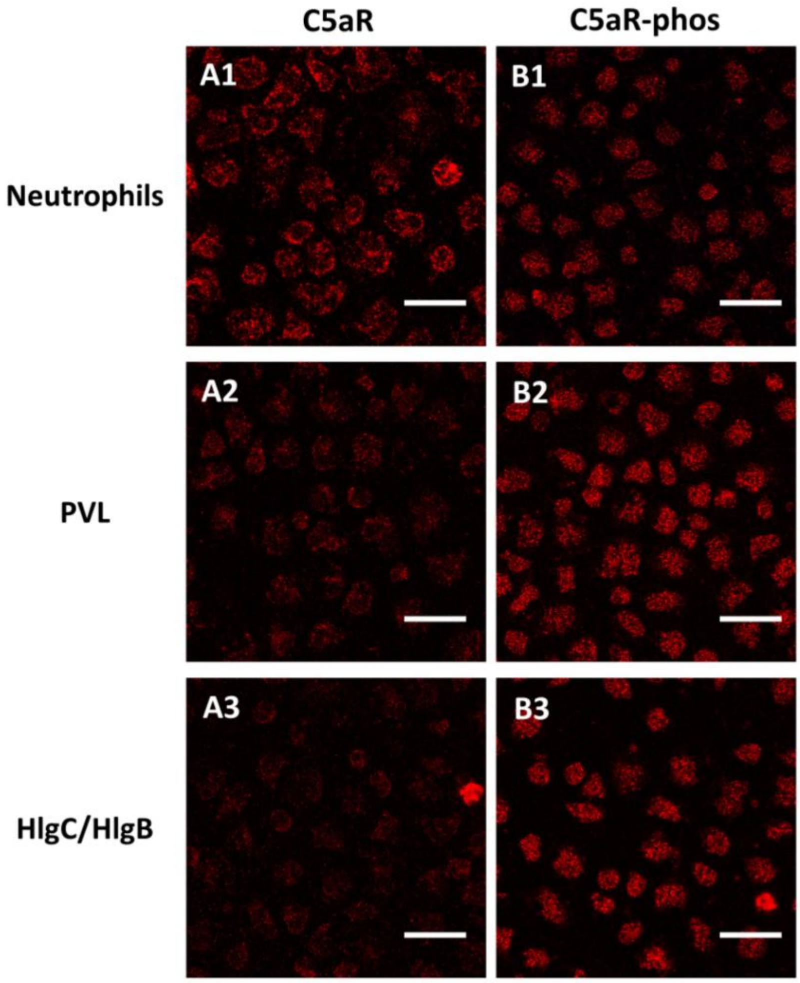

Figure S1. Binding of the PVL or HlgB/HlgC induces phosphorylation of C5aR. (**A1**) Human neutrophils stained with antibody recognize the C5aR non phosphorylated C-terminal peptide. Immunolabeling at the periphery of the cells suggests that the receptor is located near the plasma membrane; (**B1**) Human neutrophils from the same preparation stained with an antibody that recognizes the phosphorylated form of the same peptide in A1. The labeling is concentrated in the cytosol. A short 5 min incubation of human neutrophils with 2 nM PVL (**A2**,**B2**) is sufficient to reduce labeling intensity by the antibody against the non-phosphorylated receptor (**A2**) and to increase staining intensity by the antibody against the phosphorylated receptor (**B2**). Similarly, a 5 min incubation of cells with 2 nM HlgC/HlgB also reduces the labeling due to the unphosphorylated receptor antibody (**A3**) and increases staining intensity of the phosphorylated receptor antibody (**B2**). Two different experiments were conducted on 500–1000 cells. Scale bar, 20 µm. Cell Profiler pipelines were created to establish the percentage of the signal overlapping between the two fluorescent probes and measured distinct confocal microscope recording channels. Two input modules were created in the Cell Profiler cell image analysis software 2.1.1. (Windows 64-bit, revision 6c2d896). Additional information of each module is also given.
